# Protective effects of conditioned media of immortalized stem cells from human exfoliated deciduous teeth on pressure ulcer formation

**DOI:** 10.3389/fimmu.2022.1010700

**Published:** 2023-01-13

**Authors:** Yasuhiro Katahira, Fumihiro Murakami, Shinya Inoue, Satomi Miyakawa, Eri Sakamoto, Yuma Furusaka, Aruma Watanabe, Ami Sekine, Masahiko Kuroda, Hideaki Hasegawa, Izuru Mizoguchi, Takayuki Yoshimoto

**Affiliations:** ^1^ Department of Immunoregulation, Institute of Medical Science, Tokyo Medical University, Tokyo, Japan; ^2^ Department of Molecular Pathology, Tokyo Medical University, Tokyo, Japan

**Keywords:** pressure ulcer, mesenchymal stem cells (MSCs), stem cells from human exfoliated deciduous teeth-conditioned media (SHED-CM), hepatocyte growth factor (HGF), vascular endothelial growth factor (VEGF), angiogenesis, reactive oxygen species (ROS)

## Abstract

Pressure ulcers (PUs) are increasing with aging worldwide, but there is no effective causal therapy. Although mesenchymal stem cells (MSCs) promote cutaneous wound healing, the effects of the conditioned medium (CM) of MSCs on cutaneous PU formation induced by ischemia-reperfusion injury have been poorly investigated. To address this issue, herein, we first established an immortalized stem cell line from human exfoliated deciduous teeth (SHED). This cell line was revealed to have superior characteristics in that it grows infinitely and vigorously, and stably and consistently secretes a variety of cytokines. Using the CM obtained from the immortalized SHED cell line, we investigated the therapeutic potential on a cutaneous ischemia-reperfusion mouse model for PU formation using two magnetic plates. This is the first study to show that CM from immortalized SHEDs exerts therapeutic effects on PU formation by promoting angiogenesis and oxidative stress resistance through vascular endothelial growth factor and hepatocyte growth factor. Thus, the CM of MSCs has potent therapeutic effects, whereas these therapies have not been implemented in human medicine. To try to meet the regulatory requirements for manufacturing and quality control as much as possible, it is necessary to produce CM that is consistently safe and effective. The immortalization of stem cells could be one of the breakthroughs to meet the regulatory requirements and consequently open up a novel avenue to create a novel type of cell-free regenerative medicine, although further investigation into the quality control is warranted.

## Introduction

Pressure ulcers (PUs) are a type of injury that breaks down the skin and underlying tissues when an area of the skin is placed under constant pressure for a period of time. The number of patients with PUs is increasing worldwide with aging of the population (1). PUs often affect elderly patients who are confined to lying in a bed or sitting for prolonged periods of time, decreasing the quality of life (2). Current therapeutic approaches for PUs are mainly preventive measures such as frequent moving and turning of the patient’s body and use of beds and chairs designated to reduce friction on vulnerable anatomical sites. Once PUs form, only symptomatic therapies such as surgical debridement, negative pressure wound therapy, flap reconstruction, bioengineered skin substitutes, and supplementation of nutrition and growth factors to accelerate wound healing are generally used (3). Therefore, more effective causal therapy is highly desirable.

The extra pressure disrupts blood flow through the skin, and without a blood supply, the affected skin becomes starved of oxygen and nutrients and consequently ischemic. When the patient changes posture and the blood flow is reperfused to the ischemic tissue, a series of adverse events are triggered including vasculopathy, thrombosis, infiltration of inflammatory cells, production of proinflammatory cytokines, and tissue damage. Such cutaneous ischemia-reperfusion (I/R) injury is considered to be the main cause of PUs (4–6). I/R injury induces oxidative stress and endoplasmic reticulum stress, leading to cell apoptosis and formation of PUs. In accordance with the molecular mechanism of I/R injury, a simple, reproducible, and noninvasive cutaneous I/R mouse model for PUs was established using one to three I/R cycles of external application of two round magnetic plates to the dorsal skin for 12 h (ischemia) followed by release and rest for 12 h without the magnetic plates (reperfusion) (6–11).

Mesenchymal stem cells (MSCs) are adult stem cells with self-renewal, culture-expandable, and relatively non-immunogenic properties (12–14). Due to their immunomodulatory ability, multipotent differentiation capacity, and tissue repair and regeneration potential, they have garnered great interest in the field of regenerative medicine and have excellent therapeutic effects on a variety of disorders including neurological disorders, bone damage, and inflammatory and autoimmune diseases (15). Although MSCs have the ability to migrate and differentiate into damaged tissues, only a small number of surviving cells are able to migrate in a short period of time (16–19). Therefore, the main effects of MSCs may highly rely on paracrine effects by secretion of a variety of bioactive factors including cytokines, growth factors, neurotrophic factors, and exosomes (20–22). Conditioned medium (CM) or the secretome of MSCs has considerable advantages as a safer cell-free therapy than current cell transfer therapies with less concerns regarding immunogenicity, ethical regulations, and possible induction of embolism or thrombosis (23) or tumor progression (24, 25). MSCs have been shown to promote cutaneous would healing in animals and humans (26, 27), and some studies have demonstrated that they have suppressive effects on cutaneous I/R injury and subsequent skin PU formation (6, 9, 10, 28). However, to date, no study has evaluated the effects of CM from MSCs on cutaneous PU formation induced by I/R injury (29).

Although the CM or secretome of MSCs has potent therapeutic effects, it has not been implemented in human medicine because it does not meet the regulatory requirements for quality control and manufacturing that are necessary to guarantee safety and efficacy (22). To address these issues, we first established an immortalized stem cell line from human exfoliated deciduous teeth (SHED). This is an ideal cell source as it can be cultured indefinitely and stably and consistently secretes a variety of cytokines that are important for tissue repair and regeneration and immunomodulation. We investigated the therapeutic potential of CM obtained from an immortalized SHED cell line (SHED-CM) on PU formation in a cutaneous I/R mouse model using two magnetic plates. SHED-CM exerted therapeutic effects on PU formation by promoting angiogenesis and oxidative stress resistance through vascular endothelial growth factor (VEGF) and hepatocyte growth factor (HGF).

## Materials and methods

### Preparation of SHEDs from deciduous teeth

Deciduous tooth was obtained from a 10-year-old female with written informed consent given by her parent (provided by Cysay Cooperation), and SHED was prepared as described below and used for immortalization. Briefly, after washing twice with phosphate-buffered saline (PBS), the tooth was held using a forceps, and dental pulp tissue was pulled out using an endodontic file and placed in PBS containing 1% antibiotic-antimycotic solution (Sigma-Aldrich). Then the dental pulp tissue was minced and digested with collagenase and gently shaken for 1 h at 37°C. After centrifugation and passing through a nylon mesh, a single-cell suspension was cultured in Dulbecco’s Modified Eagle Medium (DMEM) supplemented with 10% fetal bovine serum (FBS) and 100 U/ml penicillin and 100 μg/ml streptomycin (Invitrogen) at 37°C in an atmosphere of 5% CO_2_/95% air. Deciduous tooth was also obtained from a 15-year-old female with written informed consent in Tokyo Medical University Hospital. SHED was similarly prepared and cultured in alpha Minimum Essential Medium (αMEM; Gibco) supplemented with 15% FBS and 100 U/ml penicillin and 100 μg/ml streptomycin and used as primary SHED. The study was approved by the institutional review board of Tokyo Medical University (SH3339, T2021-0117, T2022-0042).

### Immortalization

Individual cDNAs of human telomerase reverse transcriptase (hTERT) (30), human papillomavirus type 16 (HPV16) E6 and E7 (31), and human B cell-specific Moloney murine leukemia virus integration site 1 (BMI1) (32) with a Kozak sequence were prepared using a standard recombinant PCR technique and subcloned into the retroviral expression vector, pDON-5 Neo (Takara). Each expression vector together with pGP and pE-ampho in the Retrovirus Packing Kit Ampho were transfected into G3T-hi cells using TRansIT-293 transfection reagent according to the manufacturer’s instructions (all from Takara). After 48 h, culture supernatants were collected, centrifuged, and filtered through 0.45 µm. Then, the SHEDs were infected with the supernatants in the presence of 8 µg/ml polybrene, and 24 h later the media was replaced with fresh media containing 0.5 mg/ml G418 to obtain G418-resistant colonies. One clone (B-2) with higher proliferation abilities and secretion of cytokines was used.

### Flow cytometry

Single-cell suspensions of cells were stained with the following antibodies (all from BioLegend). MSC positive markers: FITC-conjugated anti-CD73 (clone AD2), PE-conjugated anti-CD90 (clone 5E10), APC-conjugated anti-CD105 (clone 43A3), and Brilliant Violet 510-conjugated anti-CD44 (clone IM7). Cell lineage markers: Brilliant Violet 510-conjugated anti-CD11b (clone M1/70), APC-Cy7-conjugated anti-CD14 (clone M5E2), Brilliant Violet 421-conjugated anti-CD19 (clone HIB19), Pacific Blue-conjugated anti-CD34 (clone 581), and APC-Cy7-conjugated anti-CD45 (2D1). Immunogenic markers: PE-conjugated anti-CD40 (clone 5C3), APC-conjugated anti-CD80 (clone 2D10), PE-Cy7-conjugated anti-CD86 (clone BU63), FITC-conjugated anti-HLA-DR (clone L243), and PE-Cy7-conjugated anti-HLA-A, B, C (clone W6/32). The resulting cells were analyzed using a FACSCanto II flow cytometer (BD Biosciences) and the FlowJo software application (version 10: FlowJo). Dead cells were discriminated using 7-aminoactinomycin D (7-AAD, Sigma-Aldrich). Flow cytometry gating strategy for the tetramer staining assay is shown in **Supplementary Figure 1A**.

### Cell proliferation assay

Cells (1 × 10^3^~4 × 10^4^ cells/200 μl) were incubated in the medium as indicated for 72 h, and cell proliferative activity was determined using CellTiter-Glo 2.0 Cell Viability Assay (Promega) and measured with GloMax Discovery Microplate Reader (Promega) according to the manufacturer’s instructions. To measure the proliferation-inducing ability of SHED-CM, mouse Schwann cell line IMS32 cells (2 × 10^4^ cells/200 μl) (33) (Cosmo Bio) were incubated in serum-free DMEM in the presence or absence of 30% SHED-CM for 72 h, and proliferative activity was determined as described above.

### Preparation of CM

After the SHEDs reached 70–80% confluency, they were washed twice with PBS and once with serum-free DMEM (Gibco) and then incubated in serum-free DMEM for 72 h. The culture supernatants were collected, centrifuged at 1,750 × g for 10 min to remove the cell debris, and filtered through a 0.22 µm filter. The culture supernatants were used as the CM and stored at 4°C or -80°C. We routinely used CM frozen within one year without any apparent decrease of protective effects on pressure PU formation. Once thawed, the CM were stored at 4°C while avoiding repeated freezing and thawing. Repeated treatment of freezing and thawing until three times didn’t reduce the ability of SHED-CM to induce cell proliferation, that was measured using mouse Schwann cell line IMS32 cells (33) (**Supplementary Figure 2**).

### Cytokine measurement

The content of cytokines in the CM was broadly analyzed using the Quantibody Human Cytokine Array Q1000 (RayBiotech), a multiplex enzyme-linked immunoassay (ELISA) array that can quantitatively determine the concentration of 80 different cytokines (**Supplementary Table 1**). The intensity of each signal was determined by laser scanning using GenePix 4400A (Molecular Devices).

### ELISA

Cells were washed with PBS, and resultant cells (1 × 10^5^ cells/ml) were incubated in serum-free DMEM for 72 h. The culture supernatants were collected, and concentrations of individual cytokines, VEGF, HGF, transforming growth factor beta 1 (TGF-β1), insulin-like growth factor binding protein 4 (IGFBP-4), or basic FGF (bFGF) was determined using respective sandwich ELISA kits according to the manufacturer’s instructions (R&D Systems).

### Mice

C57BL/6 mice were purchased from Sankyo Labo Service. All mice were maintained under pathogen-free conditions, and all animal experiments were approved by the President and by the Institutional Animal Care and Use Committee of Tokyo Medical University and performed in accordance with institutional, science community, and national guidelines for animal experimentation and the Animal Research: Reporting of *In Vivo* Experiments guidelines.

### Pressure ulcer mouse model

Cutaneous I/R injury was induced on the dorsal skin of mice using two magnetic plates to analyze the progress of PU formation as previously described (6). Briefly, hair on the dorsal skin was shaved 2 days before performing I/R injury. Then mice were anesthetized, and the dorsal skin was pulled up and trapped between two round ferrite magnetic plates (NeoMag) for 12 h. The magnetic plates had a 12-mm diameter and were 5-mm-thick with an average weight of 2.74 g and 1,180 G magnetic force. Twelve hours later, magnetic plates were removed and mice were rested for another 12 h. The mice developed two ulcers separated by a bridge of normal skin. For analysis, each wound site was photographed daily, and the wound areas in each photograph were measured and evaluated using FIJI (an expanded version of ImageJ, version 1.53c; National Institutes of Health) (34). To examine the effects of SHED-CM or recombinant (r) cytokines such as rbFGF, rVEGF or rHGF (all from BioLegend) on the development of PUs after I/R,100 µl SHED-CM, control medium or respective cytokines was injected into the dermis at two sites around the wound at the indicated time points.

### Exosome preparation

SHED-CM was separated into two fractions by ultracentrifugation using the Beckman Optima MAX-XP Ultracentrifuge at 49,500 rpm for 120 min at 4°C with TLA-55 rotor (Beckman Coulter): the upper fraction contained SHED-CM without exosomes and the lower fraction contained precipitated exosomes from SHED-CM. The lower fraction was resuspended in the same volume of control medium before ultracentrifugation. The content of exosomes was analyzed by western blotting and the NanoSight NS300 Nanoparticle Tracking Analysis System (Malvern Panalytical).

### Western blot analysis

The content of exosomes was analyzed by western blotting using antibodies against CD63 and CD81. Briefly, the sample was separated on an SDS-PAGE and transferred to polyvinylidene difluoride membrane (Millipore). The membrane was then blocked, probed with antibody against CD63 (clone H5C6; BioLegend) or CD81 (clone 5A6; BioLegend), followed by anti-mouse IgG conjugated to horseradish peroxidase, and visualized with the enhanced chemiluminescence detection system (Amersham Pharmacia Biotech) according to the manufacturer’s instructions. Image capture was performed with an iBright FL1500 Imaging System (Thermo Fisher Scientific).

### Immunoprecipitation

To deplete VEGF, HGF or IGFBP-4 from SHED-CM, immunoprecipitation was performed. Briefly, SHED-CM was incubated with antibody against VEGF (clone R012; Sino Biological), HGF (clone 24612; R&D Systems), IGFBP-4 (clone 82314; R&D Systems) or respective control IgG conjugated to protein G-Sepharose (GE Healthcare) overnight at 4°C. After centrifugation, the supernatant was used as SHED-CM depleted of respective cytokines. More than 90% of respective cytokines were routinely depleted compared to SHED-CM treated similarly with control IgG, that was confirmed by ELISA.

### Immunohistochemical analysis

Mouse dorsal epidermal tissues were removed, fixed in formalin, and embedded in paraffin. Deparaffinized sections (3 µm) were treated by boiling for 10 min at 121°C in 1 mM EDTA/10 mM Tris-HCl buffer (pH 9.0) for antigen retrieval of CD31 and neural/glial antigen 2 (NG2). After blocking in 2% skim milk, the sections were incubated with primary antibodies, rat anti-mouse CD31 (clone SZ31; dianova), rabbit anti-NG2 (#ab129051; abcam), or mouse anti-8-OHdG (clone N45.1; abcam), followed by incubation with HRP-labeled polymer-conjugated respective secondary antibodies (Nichirei Bioscience). Immunoreactivity was visualized with 3,3’-diaminobenzidine tetrahydrochloride (Agilent Technologies). For staining for 8-OHdG, deparaffinized sections (4 µm) were treated by autoclaving for 20 min at 105°C in pH 6 antigen retrieval buffer (PerkinElmer). Using the N-Histofine MOUSESTAIN KIT (Nichirei Bioscience), the sections were blocked and incubated with mouse anti-8-OHdG (clone N45.1; abcam), followed by incubation with HRP-labeled polymer-conjugated respective secondary antibodies according to the manufacturer’s protocol. The sections were counterstained with hematoxylin and eosin, Mayer’s hematoxylin, or Elastica van Gieson. Skin sections where cutaneous I/R injury had been induced were identified as skin lesions with trace of round magnetic plates and used for immunohistochemical analysis. Their positive areas were quantified using ImageJ (FIJI), and data were shown as relative values. Because muscular layers were non-specifically stained even in the absence of primary antibody (data not shown), these areas were omitted for quantifying positive area.

### Angiogenic activity measurement

The angiogenic activity of SHED-CM was determined using a tube formation assay (35). human umbilical vein endothelial cells (HUVECs) purchased from Lonza were cultured in maintenance medium (EBM-2 medium supplemented with EGM-2 BulletKit [Lonza]). To evaluate the performance of tube formation, HUVECs (300 µl of 4 × 10^5^ cells/ml) in EBM-2 basal medium were placed on a 289 µl Matrigel (BD Biosciences) in a 24-well plate and incubated for 8 h at 37°C. Then control medium or SHED-CM was added to the Matrigel, and photographs were taken at several time points using an optical microscope. Total tube area and branch formation rate were quantified using FIJI.

### Assessment of hypoxia/reoxygenation stress

To reproduce the I/R effect on mouse dorsal tissue, NIH3T3 cells were incubated under hypoxic conditions (1% O_2_, 5% CO_2_, and 94% N_2_) for 48 h in medium (containing 1% FBS) that had been incubated under the same hypoxic condition for 3 days. Higher concentration of FBS more greatly inhibits the effects of hypoxia on cells (36, 37). Therefore, to increase the sensitivity to hypoxia, the concentration of FBS was reduced to 1% while maintaining the cell viability. Then reoxygenation was performed by exchanging the medium with that left under normal ambient O_2_ conditions (21%) and further incubated for 6 h. Then cells were collected and analyzed for intracellular reactive oxygen species (ROS) production using the DCFDA Cellular ROS Detection Assay Kit (#ab113851; abcam) according to the manufacturer’s protocol. Briefly, 2’,7’-dichlorofluorescin diacetate (DCFDA) was diffused into cells and deacetylated by cellular esterases to a non-fluorescent compound, which then was oxidized by ROS into highly fluorescent DCF. The cells were counterstained with Hoechst 33342 (Dojindo) and detected with a confocal laser scanning microscope (FluoView FV10; Olympus). The fluorescence intensity was quantified using FIJI.

### Statistical analyses

Data are expressed as the mean ± standard error of the mean (SEM) for each group. Statistical analyses were performed using the unpaired, two-tailed Student’s *t-*test for comparisons of two groups and one-way analysis of variance with the Tukey or Dunnett multiple comparison test for comparing more than three groups using GraphPad Prism 9 (GraphPad Software). *P* < 0.05 was considered statistically significant.

## Results

### Immortalized SHEDs have potent abilities to proliferate and secrete various cytokines

**Figure 1:**
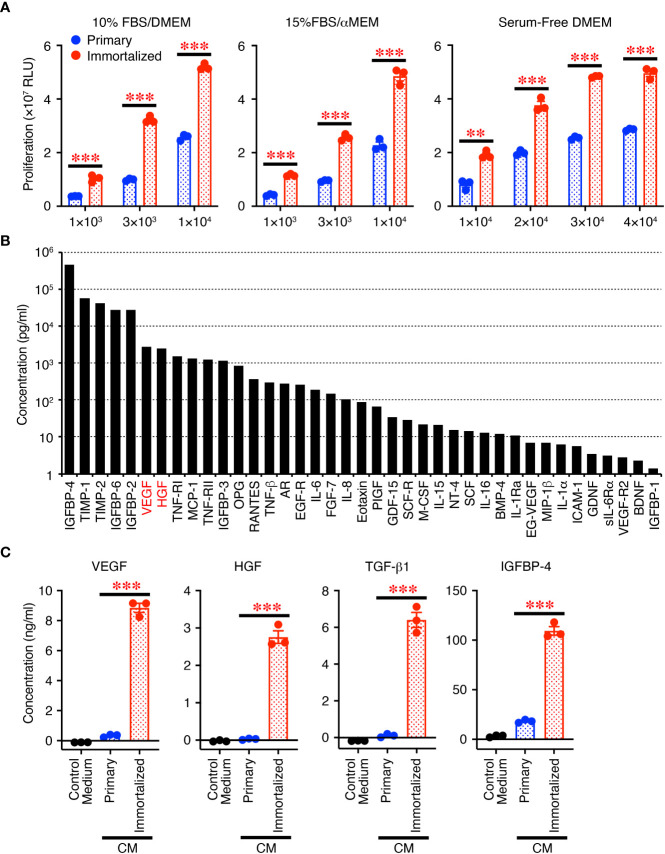
Immortalized SHEDs have enhanced abilities to proliferate and secrete various cytokines. **(A)** Immortalized SHEDs and primary SHEDs were analyzed for their proliferative activities varying the number of cells in DMEM containing 10% FBS, αMEM containing 15% FBS, and serum-free DMEM. **(B)** The content of cytokines in the CM was broadly analyzed using the cytokine antibody array, and the concentrations of 80 different cytokines were quantitatively determined. Cytokines with a concentration > 1 pg/ml are shown. **(C)** The same number of cells was cultured in serum free DMEM for 72 h, and resultant culture supernatants were analyzed for production of cytokines by ELISA. Data are shown as the mean ± SEM in triplicate and are representative of two independent experiments **(A, C)**. *P* values were determined by the unpaired two-tailed Student’s t-test. **P < 0.01, ***P < 0.001.

First, we compared the immortalized SHEDs to primary SHEDs. The immortalized SHEDs exhibited a typical MSC phenotype that is fibroblastic morphology with a bipolar spindle shape (data not shown). Cell surface analysis of the immortalized SHEDs by flow cytometry revealed that they expressed a typical pattern of cell surface markers for MSCs (12): positive for MSC markers cluster of differentiation 73 (CD73), CD90, and CD105 together with CD44; negative for cell lineage markers CD11b, CD14, CD19, and CD45; and negative for immunogenic makers CD40, CD80, CD86, and human leukocyte antigen DR (**Supplementary Figure 1B**). These patterns were similar to those of primary SHEDs, and flow cytometry showed that the purities of cells positive for typical MSC markers CD73, CD90, and CD105 were more than 99% (**Supplementary Figure 1C**). The proliferative activity of the immortalized SHEDs was much higher than that of primary SHEDs in their respective culture medium and also in serum-free medium (**Figure 1A**). Next, the cell culture supernatant in serum-free medium was collected after 72 h and analyzed for the production levels of various cytokines using a cytokine antibody array, revealing that the CM abundantly contained a variety of cytokines including growth factors and neurotrophins (**Figure 1B**). Analyses using respective specific antibody pairs for sandwich ELISA also showed that the production of cytokines such as VEGF, HGF, TGF-β1, and IGFBP-4 was much higher in the immortalized SHEDs than in primary SHEDs (**Figure 1C**). Thus, immortalized SHEDs are a useful cell source for SHED-CM, as they can be cultured indefinitely, grow vigorously, and stably and consistently secrete a variety of cytokines that are potentially important for tissue repair and regeneration and immunomodulation.

### Intradermal injection of SHED-CM shows potent inhibitory effects on PU formation

**Figure 2:**
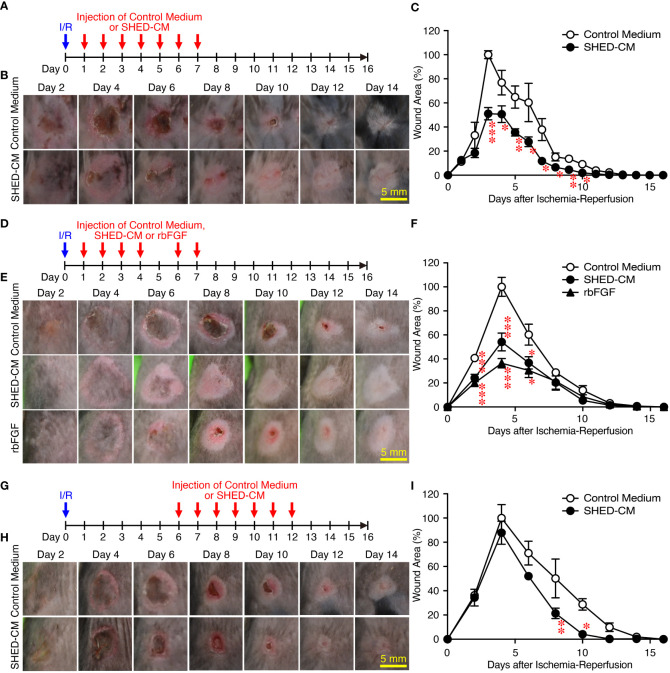
Intradermal injections of SHED-CM show potent inhibitory effects on PU formation. Cutaneous I/R injury was induced on the dorsal skin of mice using two plates to induce PU formation. After cutaneous I/R injury was performed, 100 µl SHED-CM, control medium **(A)**, or rbFGF (1 µg) in PBS **(D)** was injected into the dermis at two sites around the wound at the indicated time points. To assess the therapeutic effects of SHED-CM on PU formation, SHED-CM or control medium was injected 6 days after the cutaneous I/R injury was performed **(G)**. **(B, E, H)** Representative photographs of the PUs after induction of cutaneous I/R injury in mice that received SHED-CM or control medium. **(C, F, I)** The size of the wound areas in each photograph was evaluated using FIJI, and the relative wound areas at each time point to the maximum area in control mice on day 4 as 100% were calculated. Data are shown as the mean ± SEM (n = 5) and are representative of two **(D–I)** or more than three independent experiments **(A–C)**. *P* values were determined by the unpaired two-tailed Student’s t-test. *P < 0.05, **P < 0.01, ***P < 0.001.

Next, the effects of SHED-CM on PU formation were explored in a mouse model. PU formation was induced by generating cutaneous I/R injury by trapping the dorsal skin between two magnetic plates. The next day, SHED-CM or control medium was injected into the dermis at two sites around the wound for seven consecutive days (**Figure 2A**). The wound areas in mice injected with control medium gradually increased, peaking on day 4 and decreasing thereafter (**Figure 2B**). Although the wound areas in mice injected with SHED-CM followed similar time kinetics, they were significantly smaller on days 3 to 10 (by up to 50%) compared to mice injected with control medium (**Figure 2C**).

As an approved treatment, bFGF is used in the clinic for wound healing and scar reduction (38), as it accelerates the healing of a wide range of wounds including PUs, second degree burns, leg ulcers, and diabetic ulcers and prevents scar formation (38). The concentration of bFGF present in SHED-CM was determined to be less than 100 pg/ml by ELISA. The effect of SHED-CM was compared to that of recombinant (rbFGF; 10 µg/ml, total 1 µg each at both sites). The day after the cutaneous I/R injury was performed, SHED-CM, control medium, or rbFGF was injected into the dermis at two sites around the wound for six days as indicated (**Figure 2D**). Both SHED-CM and rbFGF showed significant inhibitory effects on PU formation, with no differences observed between them (**Figures 2E, F** **)** although the concentration of bFGF in the SHED-CM was much less than that of rbFGF. To assess the therapeutic effects of SHED-CM on PU formation, 6 days after the cutaneous I/R injury was performed on the dorsal skin, SHED-CM or control medium was injected into the dermis at two sites around the wound for seven consecutive days (**Figure 2G**). The injection of SHED-CM slightly but significantly promoted recovery from PU formation compared to control medium even at a later time (**Figures 2H, I** **)**. These results suggest that SHED-CM has potent inhibitory effects on PU formation.

### Exosomes in the SHED-CM have little inhibitory effects on PU formation

**Figure 3:**
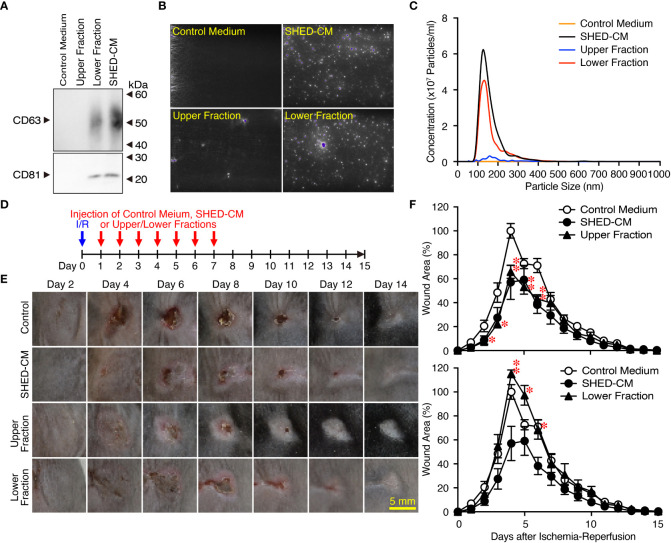
Exosomes in the SHED-CM have little inhibitory effects on PU formation. **(A–C)** SHED-CM was separated into two fractions by ultracentrifugation: the upper fraction contained SHED-CM without exosomes and the lower fraction contained precipitated exosomes from SHED-CM. The precipitate was resuspended in the same volume of control medium before ultracentrifugation. The content of exosomes was analyzed with western blotting using antibodies against CD63 and CD81 following SDS-PAGE **(A)**, and scanning electron microscopy analysis **(B)** and nanoparticle tracking analysis **(C)** with NanoSight. **(D)** After cutaneous I/R injury was induced on the dorsal skin of mice in a mouse model for PU formation using two magnetic plates, 100 µl SHED-CM, control medium, SHED-CM depleted of exosomes, and only exosomes derived from SHED-CM were injected into the dermis at two sites around the wound at the indicated time points. **(E)** Representative photographs of the ulcers are shown. **(F)** The size of the wound areas in each photograph was evaluated using FIJI, and relative wound areas at each time point to the maximum area in control mice on day 4 as 100% were calculated. Data are shown as the mean ± SEM (n = 5) and are representative of three **(A)**, three **(E, F)** or four **(B, C)** independent experiments. *P* values were determined by the unpaired two-tailed Student t-test. *P < 0.05, **P < 0.01.

SHED-CM mainly consists of proteins and extracellular vesicles such as exosomes (22, 24). Exosomes have been highlighted as a cell-cell communication and transmission tool of disease states; therefore, exosomes derived from MSCs have been applied as alternatives to MSCs for novel cell-free therapeutic strategies in a variety of disease models (39). SHED-CM was separated into two fractions by ultracentrifugation: the upper fraction contained mainly proteins and was depleted of exosomes, and the lower fraction contained precipitates of exosomes derived from the SHED-CM. The precipitate was resuspended in the same volume as that before ultracentrifugation. The purity of exosomes in each fraction was confirmed by western blotting using antibodies against exosome markers CD63 and CD81 (**Figure 3A**) and the NanoSight Nanoparticle Tracking Analysis System (**Figures 3B, C**). After cutaneous I/R injury was performed on the dorsal skin of mice to induce PU formation, 100 µl SHED-CM, control medium, SHED-CM depleted of exosomes, or exosomes derived from SHED-CM were injected into the dermis at two sites around the wound for seven consecutive days (**Figure 3D**). The upper fraction of SHED-CM depleted of exosomes showed similar inhibitory effects on PU formation as total SHED-CM, whereas the lower fraction containing exosomes derived from SHED-CM showed less inhibitory effects (**Figures 3E, F**). Thus, exosomes in SHED-CM contribute little to the inhibitory effects on PU formation.

### Inhibitory effects of SHED-CM on PU formation are highly dependent on VEGF and HGF

**Figure 4:**
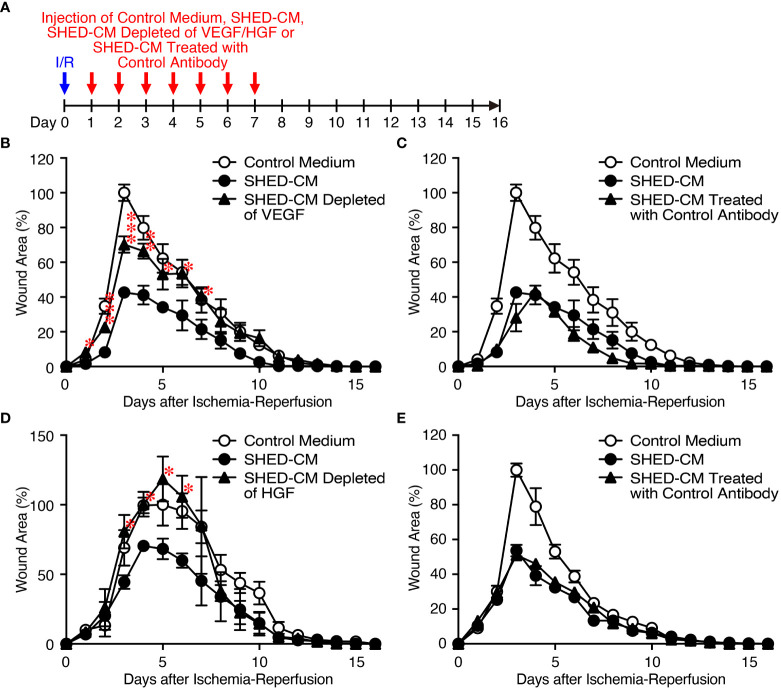
Inhibitory effects of SHED-CM on PU formation are highly dependent on VEGF and HGF. **(A)** Cutaneous I/R injury was performed on the dorsal skin of mice using two magnetic plates to induce PU formation. After cutaneous I/R injury was induced, 100 µl SHED-CM, VEGF-depleted SHED-CM **(B)**, HGF-depleted SHED-CM **(D)**, or each control antibody-treated SHED-CM **(C, E)** was injected into the dermis at two sites around the wound at the indicated time points. The size of the wound areas in each photograph was evaluated using FIJI, and the relative wound areas at each time point to the maximum area in control mice on day 3~5 as 100% were calculated. Data are shown as the mean ± SEM (n = 4~5) and are representative of two to three independent experiments. P values were determined by the unpaired two-tailed Student’s t-test. *P < 0.05, **P < 0.01, ***P < 0.001.

**Figure 5:**
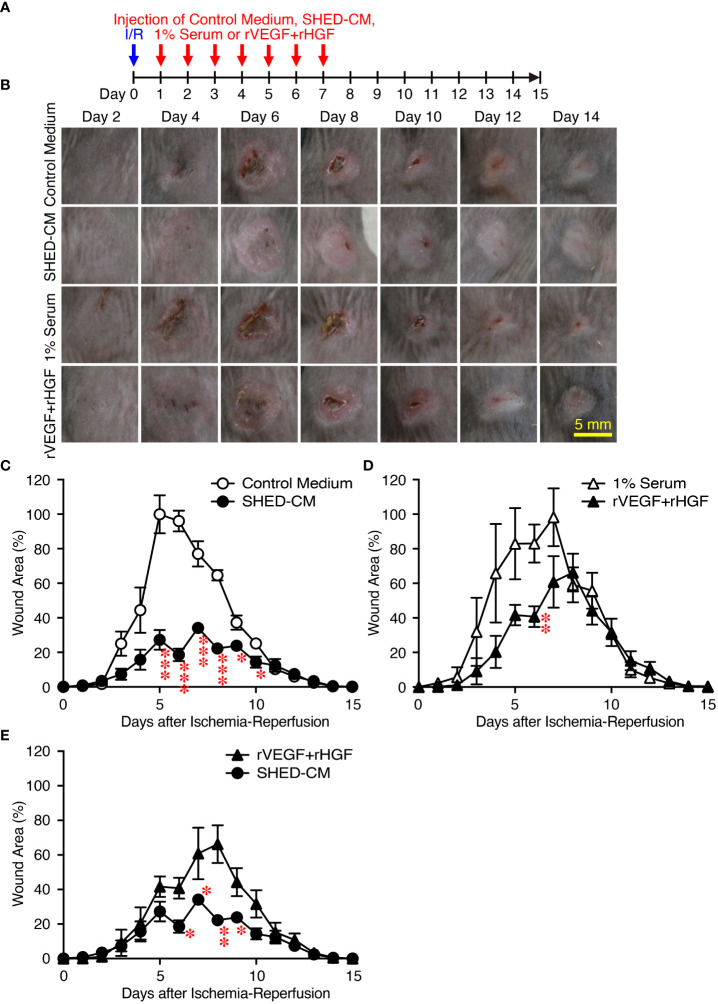
Intradermal injection of rVEGF and rHGF show only slightly inhibitory effects on PU formation. **(A)** Cutaneous I/R injury was performed on the dorsal skin of mice using two magnetic plates to induce PU formation. After cutaneous I/R injury was induced, 100 µl of a mixture of rVEGF (6 ng/ml) and rHGF (10 ng/ml), whose concentrations were similar to those present in the SHED-CM, in 1% mouse serum, 1% mouse serum alone, SHED-CM or control medium was injected into the dermis at two sites around the wound at the indicated time points. **(B)** Representative photographs of the PUs after induction of cutaneous I/R injury in mice receiving SHED-CM or control medium. **(C–E)** The size of the wound areas in each photograph was evaluated using FIJI, and relative wound areas at each time point to the maximum area in control mice on day 4 as 100% were calculated. Data are shown as the mean ± SEM (n = 3~5) and are representative of three independent experiments. *P* values were determined by the unpaired two-tailed Student’s t-test. *P < 0.05, **P < 0.01, ***P < 0.001.

Because VEGF and HGF are important for cutaneous wound healing (40, 41) and are the abundant cytokines in SHED-CM (**Figure 1B**), we assessed their inhibitory effects on PU formation using SHED-CM immunodepleted of either VEGF or HGF with their respective antibody (**Figure 4A**). ELISA analysis showed that more than 90% of VEGF or HGF in the SHED-CM was depleted compared with their respective control antibodies. Depletion of VEGF or HGF from SHED-CM with their specific antibody (**Figures 4B, D** **)** but not control antibody (**Figures 4C, E** **)** significantly reversed the inhibitory effects of SHED-CM on PU formation. Consistent with these results, injection of a mixture of rVEGF (6 ng/ml) and rHGF (10 ng/ml), whose concentrations were nearly similar to those present in the SHED-CM, inhibited PU formation (**Figure 5**). However, the inhibitory potency of the mixture was less than that of SHED-CM. Moreover, TGF-β1 was reported to promote wound healing and fibrosis (42). IGFBP-4 plays important roles in the essential cellular processes including proliferation, survival, migration, senescence, autophagy and angiogenesis, whereas the role of IGFBP-4 in cutaneous PU formation remains largely unknown (43). Our preliminary data suggested that SHED-CM immunodepleted of IGFBP-4 (approximately 90% of IGFBP-4 was depleted) seemed not to reverse the inhibitory effects of SHED-CM on PU formation (**Supplementary Figure 2**). The role of TGF-β1 in the SHED-CM remains to be determined. These results suggest that the inhibitory effects of SHED-CM on PU formation are highly dependent on VEGF and HGF but other factors are also necessary.

### SHED-CM promotes angiogenesis through VEGF and HGF

**Figure 6:**
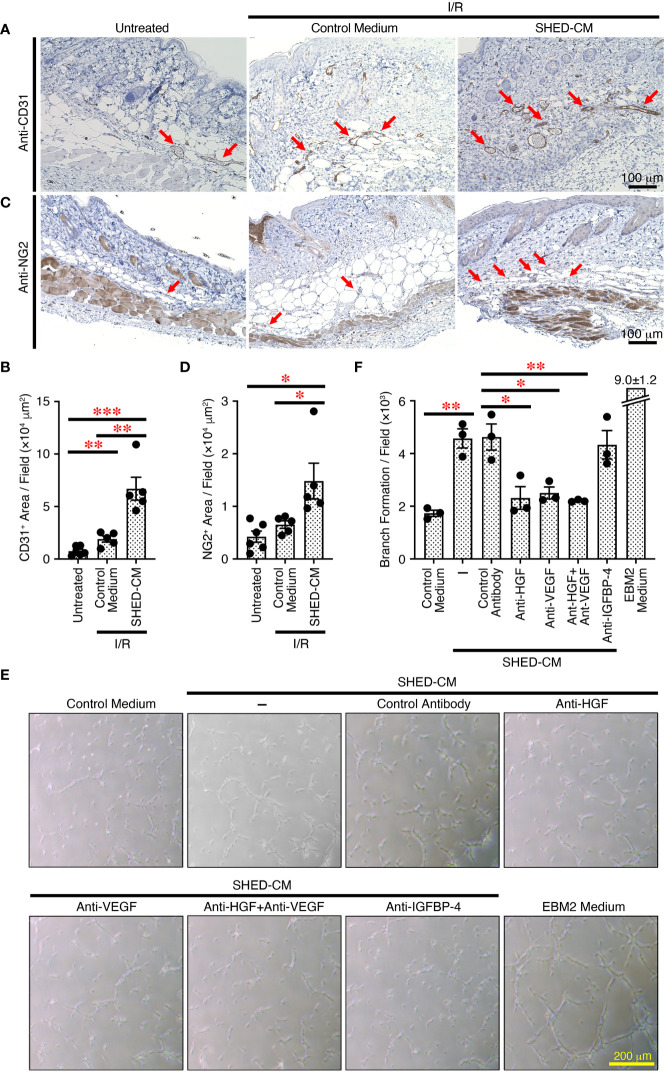
SHED-CM promotes angiogenesis through VEGF and HGF. **(A, C)** Mouse dorsal epidermal tissues of ulcers were removed on day 4 and immunohistochemically analyzed for expression of the endothelial cell marker CD31 and pericyte marker NG2 counterstaining with Mayer’s hematoxylin. Representative photographs for CD31 **(A)** or NG2 **(C)** are shown. Positive areas for CD31 or NG2 were calculated using FIJI **(B, D)**. Data are shown as the mean ± SEM (n = 5~6) and are representative of two independent experiments. **(E)** Angiogenic activities of SHED-CM, control medium, or VEGF- or HGF-depleted SHED-CM with respective antibodies, control antibodies-treated SHED-CM were determined using a tube formation assay with HUVECs. Representative photographs of the tube formation are shown. **(F)** Total tube area and branch formation rate were quantified using FIJI. Data are shown as the mean ± SEM in triplicate and are representative of five independent experiments. *P* values were determined by one-way analysis of variance with the Tukey multiple comparisons test. *P < 0.05, **P < 0.01, ***P < 0.001.

Angiogenesis or neovascularization plays a critical role in wound healing because it involves new capillary growth to form granulation tissue (44). Endothelial cells are the primary building block of blood vessels and are capable of forming tube-like structures independently, whereas endothelial cells need support from perivascular cells, pericytes, and vascular smooth muscles to form the functional vasculature, which is capable of conducting blood flow (45). Angiogenesis is important for protection against cutaneous I/R injury-induced PU formation; the number of blood vessels is decreased after cutaneous I/R injury, and injection of MSCs protects against the reduction in CD31^+^ endothelial cells (6). To explore the molecular mechanisms by which SHED-CM suppresses PU formation, immunohistochemical analysis was performed on the dorsal epidermal tissues where cutaneous I/R injury was induced on day 4, when PU formation peaks. Although a reduction in CD31^+^ endothelial cells after cutaneous I/R injury was not clearly observed, injection of SHED-CM greatly increased the number of CD31^+^ endothelial cells compared to control medium (**Figures 6A, B** **)**. In addition, a similarly increased number of NG2^+^ pericytes was observed after injection of SHED-CM compared to control medium (**Figures 6C, D** **)**. These results suggest that SHED-CM increases the numbers of CD31^+^ endothelial cells and NG2^+^ pericytes to promote angiogenesis.

To further explore the effects of SHED-CM on *in vitro* angiogenesis, we performed the tube formation assay using HUVECs. Compared to control medium, SHED-CM significantly increased the tube formation of HUVECs (**Figures 6E, F** **)**. Because VEGF and HGF are important for angiogenesis (46, 47), we examined their roles in SHED-CM in tube formation. The depletion of either VEGF or HGF from SHED-CM by immunoprecipitation with respective specific antibodies but not of IGFBP-4 or treatment with control antibody significantly decreased tube formation (**Figures 6E, F** **)**. The simultaneous depletion of both VEGF and HGF did not further inhibit tube formation (**Figures 6E, F** **)**. These results show that SHED-CM promotes angiogenesis *in vitro* and *in vivo* through VEGF and HGF.

### SHED-CM suppresses reactive oxygen species generation through VEGF and HGF

**Figure 7:**
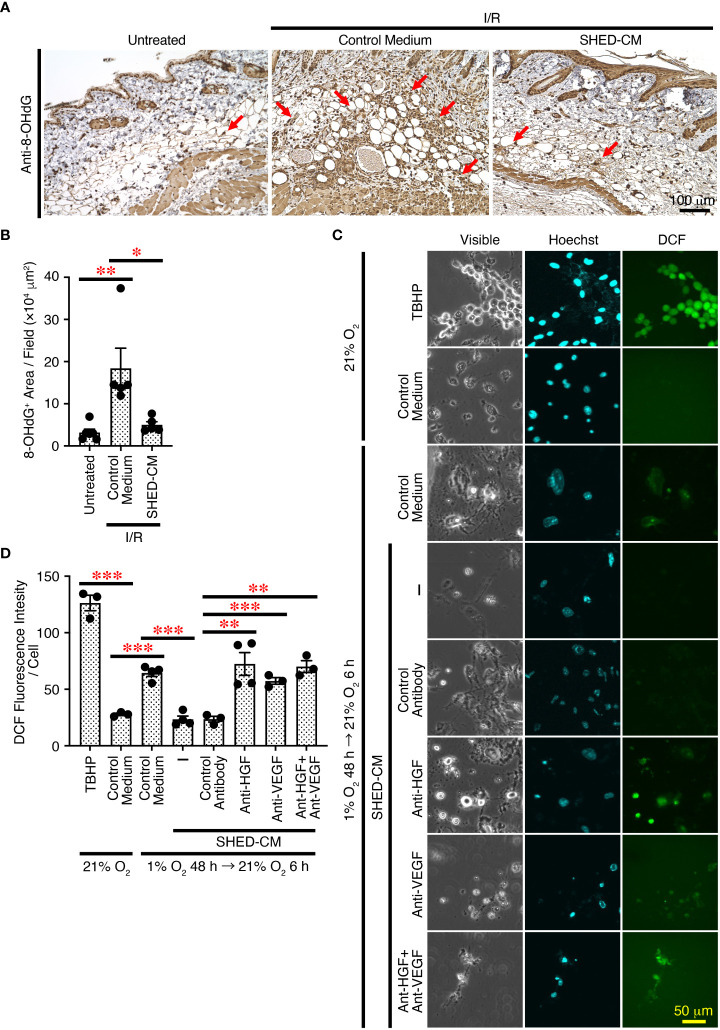
SHED-CM suppresses ROS generation through VEGF and HGF. **(A)** Mouse dorsal epidermal tissues of ulcers were removed on day 4 and immunohistochemically analyzed for expression of the oxidative stress marker 8-OHdG counterstaining with Mayer’s hematoxylin. Representative photographs for 8-OHdG are shown. Subcutaneous adipose tissue, sebaceous gland, hair bulb, dermis and epidermis were positively stained. **(B)** Positive areas for 8-OHdG were calculated using FIJI. Data are shown as the mean ± SEM (n = 5~6) and are representative of two independent experiments. **(C)** The effects of SHED-CM on ROS generation induced by hypoxia/reoxygenation *in vitro* was examined. NIH3T3 cells were incubated under hypoxic condition (1% O_2_, 5% CO_2_, and 94% N_2_) for 48 h in the medium containing 1% FBS, and reoxygenation was then performed under normal ambient O_2_ conditions (21%) in the presence of the SHED-CM, control medium, or VEGF- or HGF-depleted SHED-CM with respective antibodies, control antibodies-treated SHED-CM. After 6 h, cells were counterstained with Hoechst and analyzed for intracellular ROS production. Representative photographs of the ROS generation are shown. **(D)** The fluorescence intensity of DCF was quantified using FIJI and calculated as arbitrary unit per cell. Data are shown as the mean ± SEM in triplicate and are representative of two independent experiments. *P* values were determined by the one-way analysis of variance with the Tukey multiple comparisons test. *P < 0.05, **P < 0.01, ***P < 0.001.

Cutaneous I/R injury induces oxidative stress and resultant ROS generation, eventually leading to PU formation (4–6). ROS cause oxidative damage to proteins, lipids, and nucleic acids *in vivo*, and 8-hydroxy-2’-deoxyguanosine (8-OHdG) is one of the major products of DNA damage induced by ROS (48). To explore the molecular mechanisms whereby SHED-CM suppresses PU formation, immunohistochemical analysis was performed using the dorsal epidermal tissues of PU on day 4, when PU formation peaks. Cutaneous I/R injury greatly increased the amount of 8-OHdG^+^ damaged DNA, which was markedly reduced by injection of SHED-CM compared to control medium (**Figures 7A, B** **)**.

To further examine the effects of SHED-CM on oxidative stress *in vitro*, ROS generation was induced in NIH3T3 mouse fibroblast cells by hypoxia/reoxygenation (49). NIH3T3 cells were incubated under hypoxic conditions (1% O_2_) for 48 h in medium containing 1% FBS, and reoxygenation was performed under normal ambient O_2_ conditions (21%) in the presence of SHED-CM or control medium. After 6 h, cells were collected and analyzed for intracellular ROS production. Reoxygenation greatly increased ROS generation, which was significantly suppressed by SHED-CM (**Figures 7C, D** **)**. Because VEGF and HGF have suppressive effects on ROS generation (50–53), we examined their roles in SHED-CM on hypoxia/reoxygenation-induced ROS generation. The immunodepletion of either VEGF or HGF from SHED-CM with their respective antibody but not control antibody significantly reversed the suppressive effects (**Figures 7C, D** **)**. These results suggest that SHED-CM suppresses ROS generation *in vitro* and *in vivo* through VEGF and HGF.

### Prior intradermal injection of SHED-CM has prophylactic effects on PU formation

**Figure 8:**
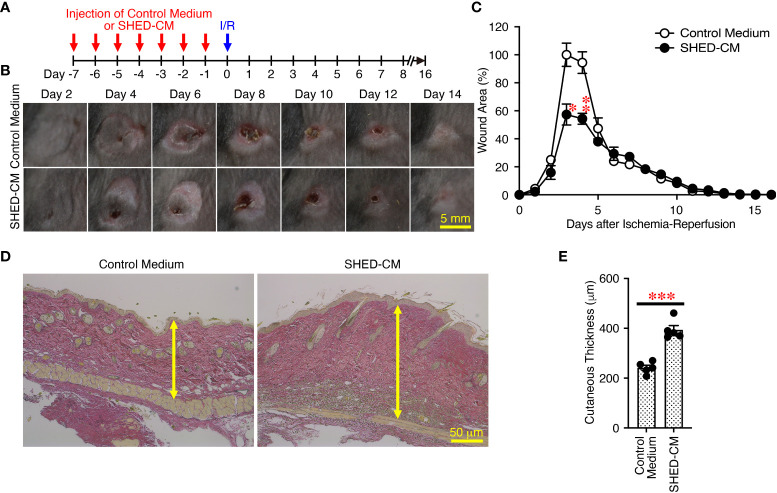
Prior intradermal injections of SHED-CM show prophylactic effects on the PU formation. **(A)** Prior to performing cutaneous I/R injury on the dorsal skin of mice using two magnetic plates to induce PU formation, mice were intradermally injected with 100 µL SHED-CM, or control medium at two sites around where the wound would emerge at the indicated time points. **(B)** Representative photographs of the ulcers after induction of cutaneous I/R injury in mice that had been received SHED-CM or control medium. **(C)** The size of wound areas in each photograph was measured and evaluated using FIJI, and relative wound areas at each time point to those in control mice on day 4 as 100% were calculated. **(D)** One day after the consecutive intradermal injections with SHED-CM or control medium for seven consecutive days, the dorsal epidermal tissues of ulcers were removed and histochemically analyzed by staining with Elastica van Gieson and counterstaining with Mayer’s hematoxylin. Representative photographs are shown. **(E)** The skin thickness was measured and evaluated in each photograph using FIJI. Data are shown as the mean ± SEM (n = 5) and are representative of two independent experiments. *P* values were determined by the unpaired two-tailed Student’s t-test. *P < 0.05, **P < 0.01, ***P < 0.001.

Finally, to further expand the applicability of SHED-CM on PU formation, the prophylactic effects of SHED-CM on PU formation were examined. Prior to induction of cutaneous I/R injury, SHED-CM or control medium was injected into the dermis at two sites on the dorsal skin, where two magnetic plates were placed, for seven consecutive days (**Figure 8A**). The day after the last injection of SHED-CM or control medium, cutaneous I/R injury was applied to induce PU formation using two magnetic plates. Prior injection of SHED-CM significantly inhibited PU formation compared to control medium (**Figures 8B, C** **)**. Histochemical analysis by staining with Elastica van Gieson for elastin, connective tissue, and collagen of the dorsal epidermal tissues obtained from mice 1 day after the last injection with SHED-CM or control medium showed that SHED-CM significantly increased the content of collagen and the cutaneous thickness compared to control medium (**Figures 8D, E** **)**. These results suggest that prior intradermal injection of SHED-CM has prophylactic effects on PU formation, possibly by increasing the content of collagen in the skin.

## Discussion

In this study, we first established an immortalized human SHED cell line and compared it to primary SHEDs. This cell line was revealed to have superior characteristics in that it grows infinitely and vigorously and stably and consistently secretes a variety of cytokines and growth factors that are potentially important for tissue repair and regeneration and immunomodulation compared to primary SHEDs (**Figure 1**). Human dental pulp tissue can be extracted from two different sources of teeth; adult permanent teeth (dental pulp stem cell, DPSC), and exfoliated baby deciduous teeth (SHED). SHED is considered to be a more immature and primitive stem cell population than DPSC and has been reported to exhibit a higher proliferative activity and differentiation capacity than DPSC (54–56), although the number of SHEDs obtained from a tooth is much less than that of DPSCs. Therefore, immortalization of SHEDs could be a reasonable approach and the resultant immortalized SHED cell line could be an ideal cell source to prepare CM for application to cell-free therapy in regenerative medicine. Using a mouse model for PU formation induced by cutaneous I/R injury with two magnetic plates on the dorsal skin of mice, intradermal injection of SHED-CM was shown to exert potent inhibitory effects on PU formation (**Figure 2**). The inhibitory effects were highly dependent on VEGF and HGF (**Figures 4**, **5**), but were unlikely to depend on IGFBP-4 (**Supplementary Figure 3**) and much less dependent on exosomes (**Figure 3**) in the SHED-CM. In addition, *in vitro* and *in vivo* analyses revealed that SHED-CM promoted angiogenesis and suppressed ROS generation in VEGF- and HGF-dependent manners (**Figures 6**, **7**). Finally, prior intradermal injection of SHED-CM showed prophylactic effects on PU formation possibly by increasing the content of collagen in the skin (**Figure 8**). This is the first study to show that CM from immortalized SHEDs exert therapeutic effects on PU formation by promoting angiogenesis and oxidative stress resistance through VEGF and HGF.

MSCs are multipotent adult stem cells that have self-renewal, culture-expandable, relatively non-immunogenic, and immunomodulatory properties, and tissue repair and regeneration potential (12–14). They can be isolated from various tissues including bone marrow, adipose tissue, umbilical cord, Wharton’s jelly, placenta, and DP, although in small amounts. MSCs have been extensively investigated and are expected to be one of the most promising therapies in the field of regenerative medicine (15). To date, numerous preclinical and clinical studies have revealed the potent therapeutic effects of MSCs on various degenerative, inflammatory, and autoimmune diseases. The underlying mechanisms are mainly considered to be homing and migration of MSCs to the injured or inflamed tissue by chemoattracting towards small chemicals or chemokines, differentiation into the damaged cells to replace them, and secretion of bioactive molecules including soluble proteins, lipids, nucleic acids, and extracellular vesicles such as exosomes (13–15). Despite the promising results, several safety issues are still a concern (57). MSCs may induce unwanted differentiation or promote tumorigenesis after transplantation, although MSCs themselves cannot form teratoma (22). Although MSCs do not express costimulatory molecules such as CD86 and CD80 or major histocompatibility complex (MHC) class II protein and are much less immunogenic and used in allogeneic transplantation (58), MSCs still express MHC class I molecules and thus are not completely invisible to the recipient’s immune system (59). Cell transfer therapy of allogeneic MSCs is strictly regulated as a regenerative medicine (60). MSCs are permissive for infections with herpes simplex virus or cytomegalovirus, and therefore have the risk of transmission of infections to the recipients (61). Because MSCs are relatively large, intravenous injection of MSCs may cause embolism or thrombosis (23). To obtain a sufficient number of MSCs for cell transfer therapy, it is generally necessary to culture and expand them *in vitro* for many weeks, although MSCs have only limited replication due to cellular senescence (62).

To overcome these issues, the CM or secretome of MSCs has garnered great interest as studies have shown that cell-free therapy using them exerts potent therapeutic effects on various diseases similarly to those observed with cell transfer therapy of MSCs (22, 24). Although the engrafting and differentiation of MSCs contribute to the therapeutic effects of cell transfer therapy, recent studies have also highlighted the paracrine effects mediated by the secretion of bioactive molecules such as cytokines, growth factors, and exosomes (25). This is because the time of period when implanted cells have been detected is short and the percentage of cells detected *in vivo* is very low (16–19). Cell-free therapy would overcome the above-mentioned drawbacks such as immune rejection, tumorigenicity, emboli or thrombosis formation, and transmission of infections. In addition, it provides more advantages over cell transfer therapy in that the handling, preservation, sterilization, and packaging are easy, and the MCS-CM can be stored for extended periods without the risk of losing its properties.

MSCs can be expanded and passaged several times but thereafter their self-renewal capacity and multipotency decline progressively due to cellular senescence (62). This is one of the major limitations for cell transfer and cell-free therapies using MSCs; therefore, MSCs have to be repeatedly established from donors through invasive extraction procedures. To overcome this issue, MSCs can be immortalized to grow vigorously and infinitely by activation of telomere (30), repression of two tumor suppressors, p53 and retinoblastoma protein (31), and modulation of the cell cycle (32), to avoid cell senescence while maintaining the mesenchymal phenotype and multipotency (63). Such immortalization can be achieved by the transduction of immortalizing genes such as hTERT (30), HPV16 E6 and E7 (31), BMI1 (32), and simian virus 40 large T antigen (64). Consistent with the present study, previous reports showed that the morphology and function of immortalized MSCs do not change after the transduction, but immortalized MSCs can rapidly and infinitely grow and secrete unlimited amounts of bioactive molecules, while maintaining their differentiation capacity and expression of the same markers of their original MSCs (65–67). Thus, the immortalization of stem cells would allow their use in therapy in large quantities and in a repeatable and consistent manner to optimize the dose and number of administrations. Therefore, MSC-CM could be evaluated for potency, dosage, and safety in a manner similar to conventional pharmaceutical agents (22). Generally, recombinant cytokines and growth factors are produced by bacterial or mammalian cells; therefore, their glycosylation is quite different from that physiologically produced in humans, resulting in reduced half-life in the blood (68). Immortalized human MSCs can make their CM or secretome more stable in blood. Thus, immortalized MSCs cannot be verified to have characteristics similar to their original normal MSCs, but rather considered to be superior to them as a source for the production of CM even in manufacturing level. Because proto-oncogenes are used to immortalize MSCs as in the present study, the most concerns of the CM of immortalized MSCs are related to safety including tumorigenicity. Notably, immortalized MSCs were demonstrated not to give rise to colonies by using a soft agar *in vitro* transformation assay (66), nor to form any tumors *in vivo* following their injection into immunodeficient mice (67), but their significant chromosomal alterations were observed (66). It was also previously demonstrated that MYC protein was present in the immortalized MSCs by introducing the proto-oncogene MYC but was not detected in the CM (69). Different from oncogenes, the presence of onco-proteins in the CM, if any, are not amplified or replicated, that reduces the risk of tumorigenesis.

Recombinant bFGF has been used in the clinic for wound healing and scar reduction over the last two decades, because preclinical and clinical research on bFGF previously revealed its potent efficacy in accelerating the healing of chronic ulcers and burn wounds in the skin (38). In this study, compared to bFGF, 100 µl SHED-CM showed similar inhibitory effects on PU formation to 1 µg rbFGF. Moreover, VEGF and HGF were demonstrated to be involved in the exertion of therapeutic effects of SHED-CM on PU formation. However, injection of a mixture of rVEGF and rHGF, whose concentrations were equally adjusted to the amounts present in the SHED-CM, showed only weak inhibitory effects on PU formation and failed to reproduce the inhibitory effects induced by total SHED-CM. These results indicate that SHED-CM, consisting of a variety of soluble molecules and exosomes, may induce synergistic inhibitory effects among these molecules on PU formation. Simultaneously, however, the fact that SHED-CM has a complex composition that may depend upon individual donors might become an impediment for regulatory approval as a regenerative medicine or pharmaceutical agent. Therefore, to try to meet the regulatory requirements for manufacturing and quality control as much as possible, it is necessary to make CM more consistently safe and effective. The immortalization of SHEDs could be one of the breakthroughs to meet the regulatory requirements and consequently open up a novel avenue to create a novel type of cell-free regenerative medicine, although further investigation into the quality control and safety is warranted.

## Data availability statement

The original contributions presented in the study are included in the article/**Supplementary Material**. Further inquiries can be directed to the corresponding author.

## Ethics statement

The studies involving human participants were reviewed and approved by The Institutional Review Board of Tokyo Medical University (SH3339, T2021-0117, T2022-0042). Written informed consent to participate in this study was provided by the participants’ legal guardian/next of kin. The animal study was reviewed and approved by The President and Institutional Animal Care and Committee of Tokyo Medical University.

## Author contributions

YK: designed and performed the experiments, analyzed data, and wrote the original draft. FM, SI, SM, and ES: performed the experiments and analyzed the data. YF, AW, and AS: performed the experiments. MK: assisted in the analysis of exosomes and immunohistochemical analysis. HH and IM: reviewed and edited the manuscript. TY: directed, conceived, designed, wrote, reviewed and edited the manuscript, and secured funding. All authors contributed to the article and approved the submitted version.
