# Supplementary material for: Protective effects of conditioned media of immortalized stem cells from human exfoliated deciduous teeth on pressure ulcer formation
Source: Front Immunol. 2023 Jan 13;13:1010700. doi: 10.3389/fimmu.2022.1010700 (PMC9881429; doi:10.3389/fimmu.2022.1010700)
Supplement: Supplementary file 1 [file DataSheet_1.pdf]

Supplementary Material

Original Research:
Protective Effects of Conditioned Media of Immortalized Stem Cells from Human Exfoliated Deciduous Teeth on Pressure Ulcer Formation

Running Title: **Immortalized SHED-CM**

Yasuhiro Katahira[1], Fumihiro Murakami[1], Shinya Inoue[1], Satomi Miyakawa[1], Eri Sakamoto[1], Yuma Furusaka[1], Aruma Watanabe[1], Ami Sekine[1], Masahiko Kuroda[2], Hideaki Hasegawa[1], Izuru Mizoguchi[1], and Takayuki Yoshimoto[1*]

[1]Department of Immunoregulation, Institute of Medical Science, Tokyo Medical University, 6-1-1 Shinjuku, Shinjuku-ku, Tokyo 160-8402, Japan
[2]Department of Molecular Pathology, Tokyo Medical University, 6-1-1 Shinjuku, Shinjuku-ku, Tokyo 160-8402, Japan

*Correspondence:
Takayuki Yoshimoto PhD, Department of Immunoregulation, Institute of Medical Science, Tokyo Medical University, 6-1-1 Shinjuku, Shinjuku-ku, Tokyo, Japan 160-8402, E-mail: yoshimot@tokyo-med.ac.jp

Supplementary Table 1. List of 80 cytokines detected by the Quantibody Human Cytokine Array Q1000 (RayBiotech).

I-309/CCL1	MCP-1/CCL2	Eotaxin/CCL11	Eotaxin-2/CCL24	MIG/CXCL9
BLC/BCA-1 /CXCL13	MIP-1α/CCL3	MIP-1β/CCL4	MIP-1δ/CCL15	RANTES/CCL5
G-CSF	M-CSF	M-CSF R	GM-CSF	SCF
SCF R	ICAM-1	IFN-γ	IL-1α	IL-1β
IL-1Ra	IL-2	IL-4	IL-5	IL-6
sIL-6Rα	IL-7	IL-8/CXCL8	IL-10	IL-11
IL-12p40	IL-12p70	IL-13	IL-15	IL-16
IL-17	TNF-α	TNF-β	sTNF RI /TNFRS1A	sTNF RII /TNFRS1B
TIMP-1	TIMP-2	BMP-4	BMP-5	BMP-7
Osteoprotegerin (OPG)	BDNF	NT-3	NT-4	β-NGF
NGF R	EGF	EGF R	HB-EGF	Amphiregulin (AR)
GDF-15	GDNF	Growth Hormone	HGF	PIGF
PDGF-AA	PDGF-BB	bFGF/FGF-2	FGF-4	FGF-7/KGF
IGF-I	IGFBP-1	IGFBP-2	IGFBP-3	IGFBP-4
IGFBP-6	TGF-α	TGF-β1	TGF-β3	EG-VEGF
VEGF	VEGR R2	VEGF R3	VEGF-D	Insulin*

*Insulin was not properly detected and therefore omitted.

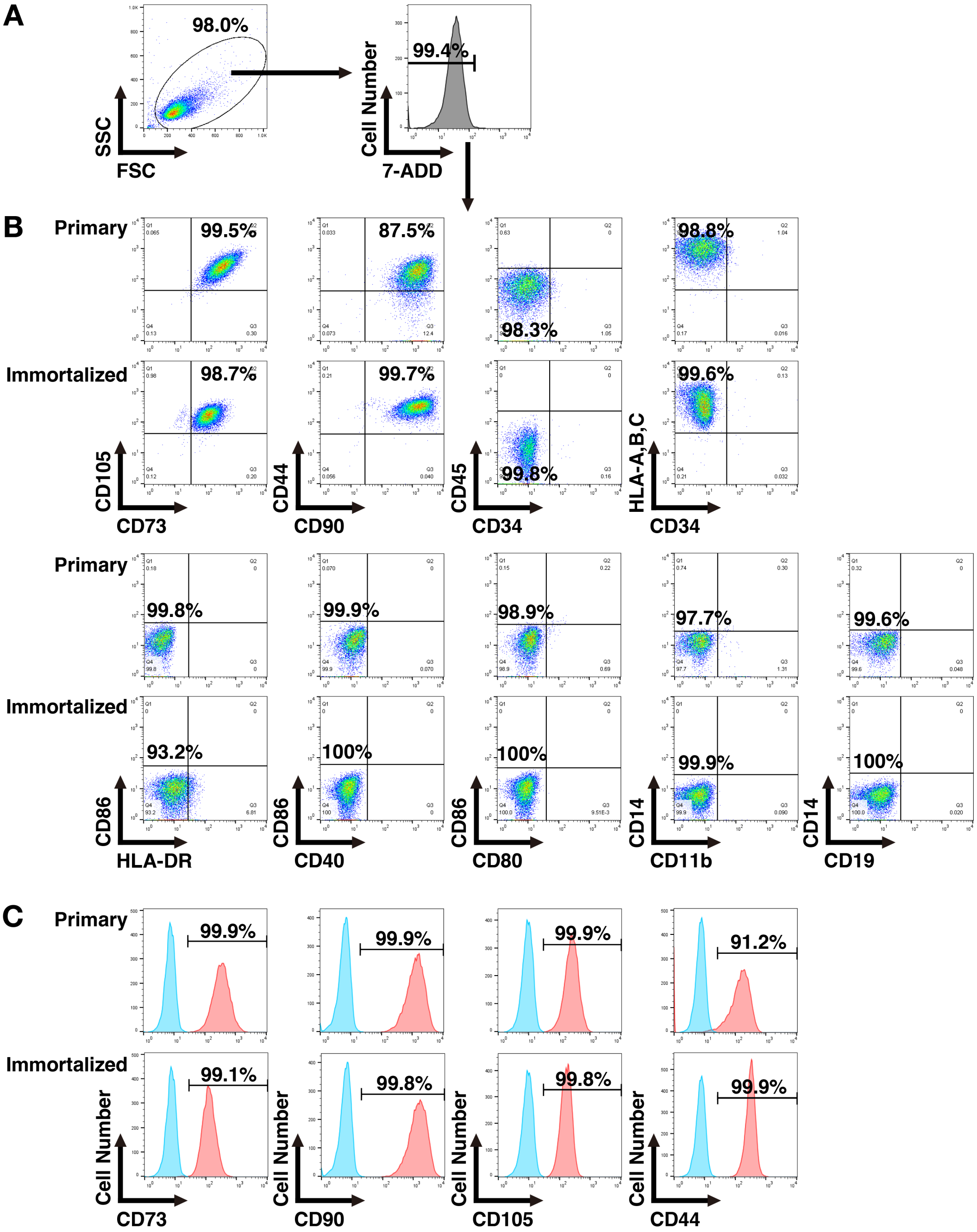

Supplementary Figure 1. Immortalized SHEDs show a typical expression pattern of cell surface makers for MSCs as primary SHEDs. **(A)** Flow cytometry gating strategy for cell surface analysis of MSCs. **(B)** Dot plot analysis of the cell surface expression on primary SHEDs and immortalized SHEDs. **(C)** Histogram analysis of the cell surface expression of MSC markers on them.

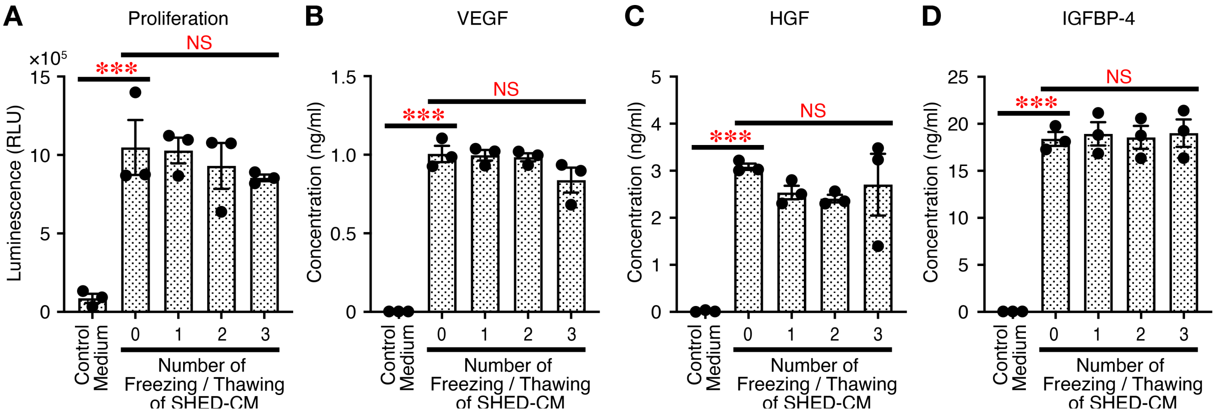

Supplementary Figure 2. Repeated treatment of freezing and thawing until three times don't reduce the ability of SHED-CM to induce cell proliferation. Freshly collected SHED-CM was frozen at -80°C in a deep freezer, thawed rapidly in water bath at 37°C, and removed from the water bath as soon as the SHED-CM was thawed. This cycle was repeated 3 times and the SHED-CM samples obtained before and after each cycle were subjected to the assay for proliferation of mouse Schwann cell line IMS32 cells **(A)** and cytokine concentration of VEGF **(B)**, HGF **(C)** and IGFBP-4 **(D)** by ELISA. Data are shown as the mean ± SEM in triplicate. *P* values were determined by one-way analysis of variance with the Dunnett multiple comparisons test. ***$P < 0.001$; NS, not significant.

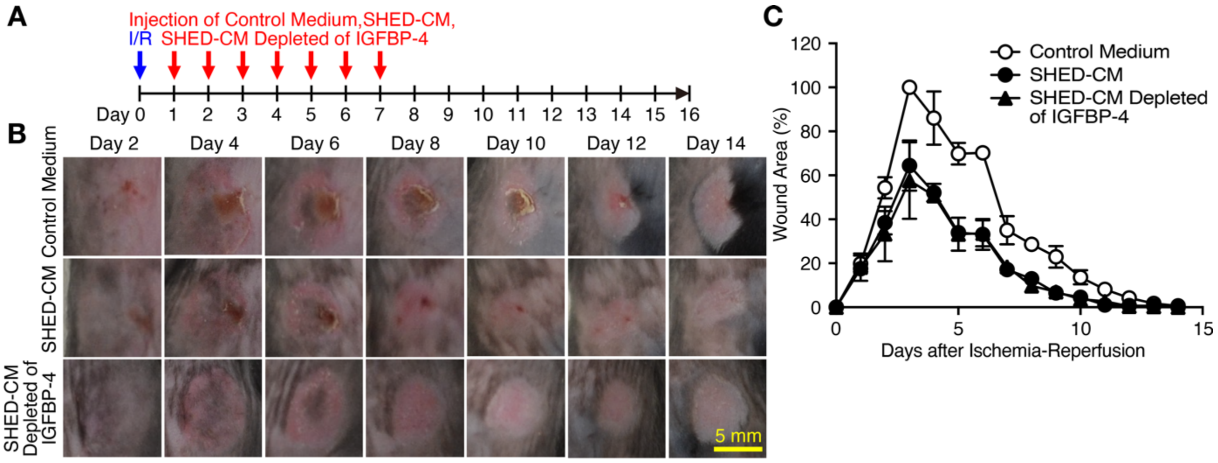

Supplementary Figure 3. Inhibitory effects of SHED-CM on PU formation are unlikely to depend on IGFBP-4. **(A)** Cutaneous I/R injury was performed on the dorsal skin of mice using two magnetic plates to induce PU formation. After cutaneous I/R injury was induced, 100 μl control medium, SHED-CM, or IGFBP-4-depleted SHED-CM was injected into the dermis at two sites around the wound at the indicated time points. **(B, C)** The size of the wound areas in each photograph was evaluated using FIJI, and the relative wound areas at each time point to the maximum area in control mice on day 4 as 100% were calculated. Data are shown as the mean ± SEM (n = 2).
